# Knowledge, perception, and attitude of Egyptian dental students toward the role of robotics and artificial intelligence in dental practices - a cross-sectional study

**DOI:** 10.1186/s12903-025-06077-0

**Published:** 2025-05-21

**Authors:** Naglaa Ezzeldin, Aya A. Salama, Karim A. Shehab

**Affiliations:** 1 Pediatric Dentistry, Faculty of Dentistry, October University for Modern Sciences and Arts, Cairo, Egypt; 2 Fixed Prosthodontics, Faculty of Dentistry, October University for Modern Sciences and Arts, Cairo, Egypt; 3 Department of Preventive Dental Sciences, College of Dentistry, Gulf Medical University, Ajman, UAE; 4 Orthodontics, Faculty of Dentistry, October University for Modern Sciences and Arts, Cairo, Egypt

**Keywords:** Undergraduate, Robot, Artificial intelligence, Dentistry, Knowledge

## Abstract

**Background:**

Rapid technological progress has made robotics (R) and artificial intelligence (AI) essential components of our everyday existence. In addition, robots designed for dental applications have been created. This study aimed to assess Egyptian dental students’ Knowledge, perception, and attitude toward the role of robotics and AI in dental practices.

**Methods:**

A cross-sectional questionnaire-based study was conducted involving 204 Egyptian students from the Faculty of Dentistry at October University for Modern Sciences and Arts. The electronic link to the questionnaire was created using Google Forms and distributed to the students via email. The questionnaire included 26 questions that assessed the students’ knowledge, perception, and attitude toward the role of R and AI in Dentistry.

**Results:**

A total of 85.8% of students were familiar with the concepts of R and AI. Among them, 66.2% demonstrated a good understanding of the applications of R and AI in dentistry, while 59.3% showed a positive attitude towards these technologies. Dental students perceived the use of R and AI favorably, particularly in dental implants and CAD/CAM technologies, with 80.6% and 82.3% expressing approval, respectively. However, 66.9% opposed the idea of replacing dentists with these technologies. Additionally, 75% expressed a desire to learn more about R and AI in the future.

**Conclusions:**

Dental students possess a good understanding of and positive attitudes toward the use of R and AI in diagnosis and interpretation. They believe that AI can play an active role in various aspects of dental practice. However, they express uncertainty about the possibility of AI and robotics replacing dentists.

**Supplementary Information:**

The online version contains supplementary material available at 10.1186/s12903-025-06077-0.

## Background

Rapid technological progress has made robotics (R) and artificial intelligence (AI) essential components of our everyday existence. R focuses on linking perception with action, where AI plays a crucial role in ensuring this connection is intelligent. Therefore, these two fields complement one another while fulfilling distinct functions. Robotics is a technological discipline that involves the design, construction, operation, and application of robots, whereas AI is a segment of computer science dedicated to creating programs that perform tasks typically requiring human intelligence. AI algorithms can address learning, perception, problem-solving, language comprehension, and/or logical reasoning [1].

AI has expanded rapidly in our everyday experiences: we rely on Google’s smart search, voice recognition in iPhone personal assistants, knowledge management, and applications like City Mapper to navigate cities effectively. ChatGPT, a prominent large language model, has recently attracted attention and is achieving global recognition [2, 3].

A report released in 2020 indicates that dental AI facilitates rapid task completion, precise diagnosis via logical decision-making, and the standardization of procedures. It is anticipated to become increasingly tailored to fulfill user requirements and enhance convenience [4].

In recent years, deep learning systems have emerged as one of the AI methods utilized for various clinical tasks. They have proven to be effective across multiple domains for automated diagnosis based on images, as well as in the design of removable partial dentures, management of temporomandibular disorders, orthognathic treatment, analysis of root morphology, assessment of periodontal diseases, detection of oral cancer, evaluation of radiolucent lesions, cephalometric analysis, and the debonding process of CAD/CAM crowns [3].

Recently, robots designed for dental applications have been created. For instance, realistic human-like robots used for training in dentistry are known as “phantoms,” while robots used for treatment encompass Simroid, nanorobots, implant robots, endo micro-robots, robotic dental drills, and orthodontic arch wire bending robots [5–7]. Dental robots have been shown to enhance the accuracy of various dental procedures across all branches of dentistry [8].

Artificial intelligence-driven simulators create realistic, interactive settings for students to practice procedures like tooth preparation and arrangement. These virtual exercises provide instant feedback, promoting skill enhancement without requiring direct patient engagement. Additionally, AI systems can assess student work, including radiographic evaluations or cavity preparations. By integrating artificial intelligence, the burden on educators can be reduced, and educational expenses can be lowered [9–11].

The increasing use of AI applications in clinical care, research, and education is evident in the growing interest in training medical students on this subject. Various organizations have suggested that healthcare workers should be educated in areas such as AI, data sourcing and safety, ethics, and the critical evaluation and interpretation of AI tools in healthcare [12]. Considering this ongoing AI transformation, it is essential to ensure that both current and future clinical professionals are informed about the capabilities of this emerging technology [7]. There is a growing recognition that dental students need to be educated about AI technologies. As a result, it will be necessary to survey dental undergraduate students to gather their opinions and views on the potential impact of AI in dentistry [13].

Amiri et al., 2024 [14] performed a systematic review and meta-analysis that offered insights into the attitudes, knowledge, and skills of medical, dental, and nursing students regarding artificial intelligence, involving 5789 participants from 24 different studies. In general, 44% of the students displayed a medium to high understanding of AI applications and principles. However, many students possessed minimal knowledge about artificial intelligence. While students exhibited moderate levels of knowledge, they typically held positive views towards AI, with 65% of all students agreeing with incorporating AI in medicine and having a favorable perspective.

Several studies conducted in Egypt [15], Saudi Arabia [16], Turkey [3], Brazil [7], India [17], and South Korea [2] have assessed dental students’ knowledge of AI. To the best of our knowledge, there is only one study that evaluates the understanding of both R and AI among dental students [8]. No study has been done about Egyptian dental students’ knowledge and attitudes regarding the employment of R technology and AI in the dental field. Therefore, this study aimed to assess Egyptian dental students’ Knowledge, perception, and attitude toward the role of robotics and artificial intelligence in dental practices. The null hypothesis was that the Egyptian students didn’t have Knowledge, perception, and attitude toward the role of robotics and artificial intelligence in dental practices.

## Methods

### Study design and study settings

A cross-sectional questionnaire-based study aimed to assess the knowledge, perceptions, and attitudes of Egyptian dental students toward robotics and artificial intelligence in the dental field. The electronic link to the questionnaire was created using Google Forms and sent to the students studying at the Faculty of Dentistry at October University for Modern Sciences and Arts via email through the university platform.

### Human ethics approval and consent to participate

The study protocol has been granted ethical approval by the Research Ethical Committee of The Faculty of Dentistry, October University for Modern Sciences and Arts, with the number REC-D 2168-4, following the Helsinki Declaration. Participants provided informed consent before starting the questionnaire by indicating their agreement or disagreement to participate in the study.

### Eligibility criteria

The study included Egyptian fourth—and fifth-year students currently studying at the Faculty of Dentistry, and both genders were involved. The exclusion criteria included students who declined to participate.

### Sampling

A nonprobability convenience sampling method was employed to distribute the questionnaire to over 453 fourth- and fifth-year students at the Faculty of Dentistry, October University for Modern Sciences and Arts [18], from September to December 2024. Of these, 204 (45%) students agreed to participate and completed the questionnaire.

### Outcomes

This study assessed Egyptian students’ knowledge, perception, and attitude toward R and AI in dentistry.

### Questionnaire design and data collection

An English-validated questionnaire from the previous study of Abouzeid et al. [8] was used. The electronic link to the questionnaire was generated using Google Forms and distributed to the students via e-mail followed by two reminder e-mails. The study participants were selected according to the eligibility criteria.

The questionnaire began with introductory paragraphs that outlined the purpose of the study, described the nature of the survey, and emphasized the voluntary nature of participation while ensuring participants’ anonymity. It also included information about the estimated time needed to complete the questionnaire, which was approximately 10 min. An informed consent was obtained from each participant by asking them to indicate whether they agreed or disagreed to participate in the study.

The questionnaire consists of 4 sections:

#### Section 1

This section collects the basic demographic information about the participants, including gender and academic grade.

#### Section 2

This section evaluates the participant’s knowledge and understanding of R and AI in dentistry. It starts by asking the participants if they are familiar with these concepts. Those unfamiliar with these concepts are not permitted to proceed further.

The section examines the participant’s understanding of the differences between R and AI, as well as their awareness of specific applications. For instance, it looks at the use of robots for vital sign monitoring, and AI for interpreting medical imaging and detecting oral cancer in its early stages.

#### Section 3

This section assesses the students’ opinions on the advantages of R and AI in dental care. These benefits include their utility in areas such as surgical assistance, orthodontics, endodontic treatments, implant planning, CAD/CAM systems, and patient data management. Furthermore, participants consider whether AI could replace dentists or contribute to education and public awareness.

#### Section 4

This section assesses participants’ attitudes toward engaging with R and AI in both professional and academic settings. The questions explore their interest in AI and robot-assisted treatments for themselves, training in simulation labs, and receiving education from teaching robots. Participants are also asked about their willingness to collaborate with robots in teams and their perspective on the importance of adopting secure digital environments for AI technology.

### Scoring knowledge, perception, and attitude

The scoring system used in this research was calculated as follows: Percentage of question score = (number of correct answers to the question / total number of answers) × 100. Each correct answer is awarded one point, while incorrect answers receive zero points.

The categorization of students based on percentage score was conducted similarly to a study established by Murali. et al., 2023 [17] participants’ knowledge was classified into four categories: Excellent for scores of 75% or higher; Good or acceptable for scores ranging from 50 to 75%; poor for scores between 25% and 49%; and scores below 25% were classified as very poor. Attitude was deemed positive for scores exceeding 50% and negative for those below 50%. Likewise, perception was defined as positive for scores above 50% and negative for scores under 50%.

### Bias

The sample selection bias was minimized by using convenience sampling to distribute the questionnaire to the students registered in the fourth and fifth years. All participants received the same explanation about the study’s nature and objectives to eliminate the information bias. To avoid reporting bias, all data were reported comprehensively, avoiding selective data reporting.

### Statistical analysis

A database was created utilizing Microsoft Excel (Microsoft; Redmond, WA, USA) and then imported into SPSS version 20 (Chicago, IL, USA) for statistical analysis. Descriptive statistical analysis, encompassing frequencies and percentages, was employed to describe the data. The Mann-Whitney test was applied to assess the relationship between unrelated samples. The association with the factors was evaluated for statistical significance using the chi-squared test, with significance defined as *p* < 0.05.

## Results

The study questionnaire was conducted with 204 participants, of whom 175 completed the entire questionnaire, resulting in a response rate of 85.8%. Twenty-nine participants, making up 14.2% of the total, were excluded from the final analysis because they answered negatively to the first ‘knowledge’ question regarding their familiarity with the concepts of R and AI in dentistry. Therefore, the final analysis included 175 participants.

### Demographic data

Out of the total of 175 students, 94 (53.7%) were females while 81 (46.3%) were males, indicating no significant difference where (*p* = 0.326). Additionally, 95 (54.3%) of the students were from the 4th year, and 80 (45.7%) were from the 5th year, also showing no significant difference where (*p* = 0.257) (Table 1).

**Table 1:** The frequencies and percentages of demographic data

Variables		Demographic data			
		n	%	Chi-square	*p*-value
**Gender**	**Female**	94	53.7%	**0.966**	**0.326ns**
	**Male**	81	46.3%		
**Grade**	**Fourth year**	95	54.3%	**1.286**	**0.257ns**
	**Fifth year**	80	45.7%		

. *; significant (*p* < 0.05) ns; non-significant (*p* > 0.05)

### Knowledge about robotics and artificial intelligence

The knowledge score of the dental students was good (65%). Out of the total students, 147 (84%) understood the differences between R and AI, ranging from a high level of knowledge to very limited knowledge. Additionally, 80.6% of the students exhibited a good knowledge of how to use R and AI in examinations and their interpretations. Furthermore, 73.1% of the students recognized the applications of R and AI in the diagnosis of tissue samples (Table 2).

**Table 2:** The frequencies and percentages of knowledge about R and AI:

Variables		knowledge about robotics and artificial intelligence			
		*n*	%	Chi-square	*p*-value
**Q2**	**Yes** ^**†**^	72	41.1%	**23.737**	< 0.001*
	**No**	28	16%		
	**Very little**	75	42.9%		
**Q3**	**Yes** ^**†**^	100	57.1%	**45.680**	< 0.001*
	**No**	43	24.6%		
	**Don`t know**	32	18.3%		
**Q4**	**Yes** ^**†**^	122	69.7%	**105.269**	< 0.001*
	**No**	21	12%		
	**Don`t know**	32	18.3%		
**Q5**	**Yes** ^**†**^	141	80.6%	**175.760**	< 0.001*
	**No**	16	9.1%		
	**Don`t know**	18	10.3%		
**Q6**	**Yes** ^**†**^	128	73.1%	**125.840**	< 0.001*
	**No**	18	10.3%		
	**Don`t know**	29	16.6%		
**Q7**	**Yes** ^**†**^	120	68.6%	**104.994**	< 0.001*
	**No**	13	7.4%		
	**Don`t know**	42	24%		
**Total**	**Yes**	65%		**486.554**	< 0.001*
	**No**	13.2%			
	**Very little/Don`t know**	21.7%			
**Total knowledge score**: **Mean**		0.65			
**SD**		0.28			
**Median (Range)**		0.67 (1.00)			

*; significant (*p* < 0.05) ns; non-significant (*p* > 0.05)

†; correct answer

### Perception of robotics and artificial intelligence

The dental students showed A positive perception toward R and AI (71%). Additionally, 80.6% of students held a favorable perception of the role of R and AI in implant placement and 82.3% had a positive perception of R and AI in CAD/CAM processes. However, 66.9% of students opposed the idea of replacing dentists permanently with R and AI (Table 3).

**Table 3:** The frequencies and percentages of perception to R and AI:

Variables		Perception toward robotics and artificial intelligence			
		*n*	%	Chi-square	*p*-value
**Q8**	**Yes** ^**†**^	126	72%	**124.949**	< 0.001*
	**No**	10	5.7%		
	**Don’t know**	39	22.3%		
**Q9**	**Yes** ^**†**^	114	65.1%	**79.691**	< 0.001*
	**No**	31	17.7%		
	**Don`t know**	30	17.1%		
**Q10**	**Yes** ^**†**^	135	77.1%	**152.377**	< 0.001*
	**No**	14	8%		
	**Don`t know**	26	14.9%		
**Q11**	**Yes** ^**†**^	94	53.7%	**32.789**	< 0.001*
	**No**	42	24%		
	**Don’t know**	39	22.3%		
**Q12**	**Yes** ^**†**^	141	80.6%	**179.874**	< 0.001*
	**No**	6	3.4%		
	**Don`t know**	28	16%		
**Q13**	**Yes** ^**†**^	144	82.3%	**190.640**	< 0.001*
	**No**	8	4.6%		
	**Don`t know**	23	13.1%		
**Q14**	**Yes**	39	22.3%	**91.931**	< 0.001*
	**No** ^**†**^	117	66.9%		
	**Don’t know**	19	10.9%		
**Q15**	**Yes** ^**†**^	141	80.6%	**176.583**	< 0.001*
	**No**	12	6.9%		
	**Don`t know**	22	12.6%		
**Q16**	**Yes** ^**†**^	108	61.7%	**64.469**	< 0.001*
	**No**	28	16%		
	**Don’t know**	39	22.3%		
**Total**	**Yes**	66.2%		**763.691**	< 0.001*
	**No**	17%			
	**Don`t know**	16.8%			
**Total perception score**: **Mean**		0.71			
**SD**		0.25			
**Median (Range)**		0.78 (1.00)			

*; significant (*p* < 0.05) ns; non-significant (*p* > 0.05)

†; correct answer

### Attitude toward robotics and artificial intelligence

The dental students showed A positive attitude toward R and AI (71%). When examining students’ attitudes towards the treatment provided by R and AI, as well as the treatments they received from these technologies, there were no significant differences in their acceptance or rejection of the concept. The *p*-values for acceptance and rejection were 0.112 and 0.705, respectively. Of the students surveyed, 31.4% accepted the idea of receiving lectures or workshops from a robot, while approximately 23% neutrally accepted it. Additionally, 32% believed that these robotic teaching methods would increase their self-confidence more than traditional teaching methods, and 23.4% naturally felt the same (Table 4).

**Table 4:** The frequencies and percentages of attitude toward R and AI:

Variables		Attitude toward robotics and artificial intelligence			
		*n*	%	Chi-square	*p*-value
**Q17**	**Yes** ^**†**^	98	56%	**2.520**	**0.112ns**
	**No**	77	44%		
**Q18**	**Yes** ^**†**^	90	51.4%	**0.143**	**0.705ns**
	**No**	85	48.6%		
**Q19**	**Yes** ^**†**^	127	72.6%	**35.663**	< 0.001*
	**No**	48	27.4%		
**Q20**	**Yes** ^**†**^	55	31.4%	**14.000**	0.001*
	**No**	80	45.7%		
	**Neutral**	40	22.95		
**Q21**	**Yes** ^**†**^	56	32%	**11.874**	0.003*
	**No**	78	44.6%		
	**Neutral**	41	23.4%		
**Q22**	**Yes** ^**†**^	103	58.9%	**51.337**	< 0.001*
	**No**	35	20%		
	**Neutral**	37	21.1%		
**Q23**	**Yes** ^**†**^	131	74.9%	**137.017**	< 0.001*
	**No**	16	9.1%		
	**Neutral**	28	16%		
**Q24**	**Yes** ^**†**^	120	68.6%	**98.000**	< 0.001*
	**No**	25	14.3%		
	**Don`t know**	30	17.1%		
**Q25**	**Yes** ^**†**^	129	73.7%	**129.646**	< 0.001*
	**No**	17	9.7%		
	**Don’t know**	29	16.6%		
**Q26**	**Yes** ^**†**^	128	73.1%	**126.251**	< 0.001*
	**No**	17	9.7%		
	**Don’t know**	30	17.1%		
**Total**	**Yes**	59.3%		**579.848**	< 0.001*
	**No**	27.3%			
	**Neutral/Don’t know**	13.4%			
**Total attitude score**: **Mean**		0.59			
**SD**		0.28			
**Median (Range)**		0.60 (1.00)			

*; significant (*p* < 0.05) ns; non-significant (*p* > 0.05)

†; correct answer

### The relations

There was no statistically significant difference between males and females regarding knowledge, perception, and attitude scores, with *p*-values of 0.367, 0.973, and 0.858, respectively (Table 5). Furthermore, no statistically significant differences were observed in knowledge and perception scores between students in their 4th and 5th years. However, there was a statistically significant difference in attitudes score, with 5th-year students achieving the highest mean score (0.63), *p* = 0.033 (Table 6).

**Table 5:** The relation between male and female to the knowledge, perception, and attitude

Variables	Robotics and Artificial intelligence								
	Males				Females				*p*-value
	Mean	SD	Median	Range	Mean	SD	Median	Range	
**knowledge**	0.67	0.28	0.67	1.00	0.63	0.29	0.67	0.83	**0.367ns**
**Perception**	0.71	0.26	0.78	1.00	0.71	0.24	0.78	1.00	**0.973ns**
**Attitude**	0.60	0.29	0.60	1.00	0.59	0.27	1.60	1.00	**0.858ns**

ns; non-significant (*p* > 0.05)

**Table 6:** The relation between fourth- and fifth-year students to the knowledge, perception, and attitude

Variables	4th grade				5th grade				*p*-value
	Mean	SD	Median	Range	Mean	SD	Median	Range	
**knowledge**	0.59	0.29	0.67	1.00	0.68	0.23	0.67	1.00	**0.360ns**
**Perception**	0.69	0.26	0.78	1.00	0.76	0.16	0.78	0.78	**0.812ns**
**Attitude**	0.53	0.24	0.60	1.00	0.63	0.30	0.70	1.00	0.033*

*; significant (*p* < 0.05) ns; non-significant (*p* > 0.05)

## Discussion

The fields of medicine and dentistry are experiencing rapid technological advancements due to the use of AI. AI has demonstrated its effectiveness as a valuable tool in various aspects of healthcare, with the potential to improve patient care. Improving the efficiency of healthcare systems can result in better health outcomes. Integrating AI into dental education is essential for preparing future dentists for the changing healthcare landscape [19].

The dental field is advancing significantly due to extensive research and development in R and AI. Robotics focuses on turning perception into action, making these two technologies complementary yet distinct in their purposes [8]. The current study aims to evaluate Egyptian dental students’ knowledge, perceptions, and attitudes toward using R and AI in dentistry.

In this study, the selected sample consisted of dental students, as the younger dentists are likely to be more comfortable with using technology and more inclined to support the integration of AI and related technologies in clinical practice. They believe these advancements can improve the quality of work and make procedures easier than traditional dental methods. Additionally, they see the availability of such technologies as prestigious and trustworthy in the clinic [20]. It remains uncertain if the current curriculum is preparing dental students for the integration of R and AI in their future practices [21].

In the presented study, 53.7% of the participants were female, while 46.3% were male. This distribution is consistent with the findings of Yüzbaşıoğlu, 2020 and Elchaghaby and Wahby, 2025. The study participants were 54.3% of fourth-year students and 45.7% of fifth-year students. Several studies have included students from various grades; for instance, Elchaghaby and Wahby, 2025 selected students from the third, fourth, and fifth years, while Yüzbaşıoğlu, 2020 included students from all five grades [3, 15]. In this study, only fourth and fifth-year students were selected because, according to the Egyptian curriculum, they are considered clinical-year students and are knowledgeable about the theoretical and clinical aspects of different dental fields.

Among the study participants, 85.8% were familiar with R and AI technologies in dentistry. This finding is consistent with the results of Yüzbaşıoğlu 2020 and Abouzeid et al. 2021, who found that 78.9% and 83.7% of the participants, respectively, were familiar with AI technologies in dentistry [3, 8]. This consistency may refer to the fact that both study participants were students, and the younger generations are more well-acquainted with recent technologies.

Most of the study participants had heard about AI and robotics in dentistry; however, only 41% could differentiate between the two. This finding is consistent with the results of Krishnaprakash et al., 2023 [22], who found that half of the participants understood the distinction between the technologies. Conversely, only 7% of Saudi students in the study by Abouzeid et al., 2021 [8] were able to identify the differences. These discrepancies may stem from differences in educational curriculum between various countries.

About 57% of the students indicated that R could aid in diagnosis and treatment planning, while 69.7% recognized its role in measuring patients’ vital signs. These findings align with those of Abouzeid et al. 2023, who reported that 60.6% had similar insights [8]. According to Elchaghaby and Wahby, 2025, 49.7% of students believe that AI will be helpful in diagnosis, while 24.7% think that AI will be beneficial in treatment plans. Although this study was also conducted with Egyptian students, the lower results in the Elchaghaby and Wahby study may be attributed to the fact that 21% of the participants were in their third year, which could indicate that they have less knowledge compared to senior students.

Misdiagnosis poses significant challenges in dental practice, as it can compromise the quality of care provided to patients. Subtle changes in a patient’s condition may often go unnoticed by clinicians [17]. In this study, the participants reported the effectiveness of AI in areas such as examination and interpretation, pathology, and tissue sampling. Additionally, they noted AI’s role in detecting early-stage oral cancer, with percentages of 80.6%, 73.1%, and 68.6% respectively. These results followed Egyptian, Turkish, and Indian students [3, 15, 17]. The results demonstrate dental students’ interest in new technologies like AI and their eagerness to learn.

When study participants were asked if the use of R and AI is beneficial in dentistry, 72% of students expressed that they found AI and robotics exciting. Students from Egypt, Turkey, and Saudi Arabia expressed enthusiasm for these technologies, with 57%, 51%, and 47.3% respectively [3, 8, 15]. The lower responses in these studies may be due to differences in countries and the grades of the selected students, in addition to a shortage of attendance at workshops and conferences related to AI.

Several students from different countries agreed that AI could be utilized across all branches of dentistry, particularly in surgery, prosthodontics, orthodontics, restorative treatments, and implantology [3, 8, 15, 23]. This consensus was also shared by the Egyptian students involved in this study. This may be due to students viewing robots as an appealing option for patient care in the clinics. They might believe that having a robot on-site would give patients the impression that the dentist is highly advanced and utilizes the latest technology [20].

The application of surgical robots for tasks such as bone milling, drilling holes, making deep saw osteotomy cuts, and performing orthognathic surgery shows great promise. Micro endodontic robots have the potential to safely and accurately treat patients with root canal issues in and reliable manner. This technology could help reduce dependence on individual dentists’ skills and minimize human error [24].

A total of 66.9% of participants rejected the idea that R and AI could permanently replace dentists, which aligns with several studies [2, 3, 8, 15]. Deloitte’s partnership with the Oxford Martin Institute indicates that AI may be able to automate 35% of jobs in the UK within the next 10 to 20 years. Nevertheless, replacing human positions with AI in the healthcare sector is more complicated because of the ethical concerns related to its application in this area [25].

Unlike many other professions, it is evident that AI technology will have difficulty fully replacing medical professionals such as physicians or dentists. This is due to several factors, including the inability of AI to engage in meaningful conversations with patients, which are essential for building trust, providing reassurance, and showing empathy. Furthermore, while sensors can gather crucial data to support diagnosis, doctors will always be necessary to interpret complex cases, integrate patients’ medical histories, perform physical examinations, and engage in ongoing discussions with patients [26, 27].

A total of 56% of the students recommend treatment administered by robots, while 51% are willing to undergo such treatment themselves. These findings align with the studies conducted by Abouzeid et al., 2021 Krishnaprakash et al., 2023 and Alzahrani, 2024 [8, 20, 22], However, Abouzeid et al. reported higher rates of recommendation and acceptance, at 83% and 84.5%, respectively. These discrepancies may be attributed to differences in countries, educational curriculum, and the characteristics of the study participants. Notably, approximately 41% of the participants in Abouzeid et al.‘s study were interns and postgraduate students, who tend to have more experience and familiarity with AI technology compared to undergraduate students.

The application of R and AI in educational tools is rapidly growing, and the heightened enthusiasm among study participants to learn from robots is evident, as more than 50% of the students responded positively or neutrally about utilizing the robot simulation lab for training in restorative treatment, attending a lecture, or participating in a workshop led by a robot. Additionally, they concurred that receiving information from a teaching robot boosts their self-confidence, a conclusion supported by Abouzeid et al., 2021, and Krishnaprakash et al., 2023 [8, 22].

It is crucial to pay particular attention to R and AI in education, as an articulated curriculum should be established to enhance the role of robotics in teaching. Tailored curricula, educational resources, and training programs for teachers should be designed for various types of robotic technology and different levels of dentistry education [8].

Over 60% of the participants at the university are open to using R and AI in their work. They believe that incorporating these technologies could improve their clinical practice and emphasize the necessity of transitioning to a secure digital environment that leverages AI applications. Furthermore, they advocate for the development of a healthcare system that utilizes the latest technologies. These findings are consistent with the research of Abouzeid et al. 2021 and Krishnaprakash et al., 2023 [8, 22].

Almost 75% of students express interest in learning about R and AI in the future. Since education is a continuous process, it is essential to improve the curriculum and enhance the overall educational experience. the integration of R and AI in the dental field. Additionally, there should be a focus on the utilization of these technologies during dental workshops and conferences.

In this study, no significant differences were found between males and females regarding their knowledge, perception, and attitude toward the subject. These findings align with those of Elchaghaby and Wahby 2025 [15]. A possible explanation for this outcome is that both genders are studying the same curriculum.

However, there was a significant difference in attitudes toward the utilization of R and AI in dentistry based on students’ years of study. Fifth-year students exhibited a more positive attitude, which is consistent with the results of Yüzbaşıoğlu, 2020. This could be attributed to the fact that senior students are generally less apprehensive about new technologies [3]. The null hypothesis was rejected because the study participants demonstrated good knowledge, positive perception, and attitude.

Ultimately, it is important to emphasize the need for incorporating such topics into dental programs, along with proposing strategies to address the potential limitations of technology. Advocating for research and development of R and AI within dental education and its curriculum, as well as involving all stakeholders in the development process while ensuring a solid legal and ethical foundation, will be crucial for the success of the R and AI sector.

### Limitations

This study had certain limitations. Firstly, the responses from dental students might not accurately reflect their actual knowledge and practices, as they may aim to present themselves as more competent individuals in their field. This could potentially result in the emergence of social desirability bias.

The research was limited to Egypt, so varying educational curriculum and teaching methods in other countries may influence the findings. Furthermore, the participants chosen may not have adequately represented the overall population of dental students, given the low response rate to the Google form. Additionally, the questionnaire relied on self-reported data, which may not apply to all dental students in Egypt.

Furthermore, since we focused solely on the attitudes of undergraduate dental students, it is possible that postgraduate students and even more experienced dentists do not share the same more optimistic perspectives.

## Conclusions

Dental students possess a good understanding of and positive attitudes toward the use of R and AI in diagnosis and interpretation. They believe that AI can actively participate in various aspects of dental practice, including surgery, restorative treatment, and orthodontics. However, they are skeptical about the possibility of AI and robotics replacing dentists.

The results offer important perspectives on the obstacles that AI researchers need to tackle and the potential applications of AI in dentistry. Future studies are recommended to encompass the entire nation to provide a more comprehensive understanding of robotics and AI among postgraduate students and dental practitioners.

## Electronic supplementary material

Below is the link to the electronic supplementary material.

Supplementary Material 1

Supplementary Material 2

## Data Availability

All data generated or analyzed during the study are included in this research article.
