# Supplementary material for: Knowledge, perception, and attitude of Egyptian dental students toward the role of robotics and artificial intelligence in dental practices - a cross-sectional study
Source: BMC Oral Health. 2025 May 21;25:747. doi: 10.1186/s12903-025-06077-0 (PMC12093707; doi:10.1186/s12903-025-06077-0)
Supplement: Supplementary file 1 — Supplementary Material 1 [file 12903_2025_6077_MOESM1_ESM.docx]

**Knowledge, perception, and attitude of Egyptian dental students toward the role of robotics and artificial intelligence in in dental practices - A cross-sectional study**

**Section Ⅰ: Demographic information:**

Gender:

- Male
- Female

Grade:

- 4^th^ year
- 5^th^ year

**Section ⅠⅠ: knowledge about robotics and artificial intelligence:**

1. Have you heard about artificial intelligence and robotics in dentistry?

- Yes
- No

(If the answer is ‘no’, do not proceed with the questionnaire)

2. Do you know the difference between artificial intelligence and robotics?

- Yes
- No
- Very little

**Q. Mark your answer to the following statements:**

| **Questions/Statement** | **Yes** | **No** | **I don’t know** |
| --- | --- | --- | --- |
| 3. Robot technology is used to assist with patient diagnosis and the development of an integrated treatment plan. |  |  |  |
| 4. Robot are used in measurement of vital signs such as pulse, breathing, temperature, blood pressure and ECG. |  |  |  |
| 5. Artificial intelligence is used in examinations and their interpretation, e.g. radiographs, CBCT, MRI, differentiation between vital and pathological signs. |  |  |  |
| 6. Artificial intelligence is used in the field of pathology for accurate reading of tissue samples and assists in the diagnosis. |  |  |  |
| 7. Artificial intelligence is used in detection of oral cancer in its early stages, such as during health campaigns. |  |  |  |

**Section ⅠⅠⅠ: Perception to robotics and artificial intelligence:**

**Give your opinion on the following statements:**

| **Questions/Statement** | **Yes** | **No** | **I don’t know** |
| --- | --- | --- | --- |
| 8. Is R/AI use in dentistry beneficial? |  |  |  |
| 9. Automated surgical robots in oral and maxillofacial surgery work with the surgeon to perform a certain operation or may act as a surgeon’s assistant. |  |  |  |
| 10. In the field of orthodontics, artificial intelligence can provide a more accurate digital view of the mouth than the traditional method, and predict the movement and the treatment of teeth, and work applications with wire rather than the laboratory. |  |  |  |
| 11. In endodontic treatment, working robots may reduce possible treatment errors and increase the quality of treatment. |  |  |  |
| 12. R/AI may contribute to predicting the correct placement of dental implants, providing 3D views before and during the process through an integrated simulation system. |  |  |  |
| 13. R/AI facilitates CAD/CAM and process of teeth arrangement |  |  |  |
| 14. Can artificial intelligence replace dentists permanently? |  |  |  |
| 15. Artificial intelligence facilitates the preservation of patient information, data and accessibility quickly and accurately. |  |  |  |
| 16. Can robots contribute to increased career production, medical education and awareness in the community and individuals? |  |  |  |

**Section ⅠV: Attitude toward robotics and artificial intelligence:**

17. Would you recommend treatment done with R/AI?

- Yes
- No

18. Would you prefer treatment with R/AI done on yourself, if needed?

- Yes
- No

19. Would you prefer to work in the robot simulation lab for training in endodontics, crowns, bridges and fillings etc.?

- Yes
- No

20. Would you prefer to receive lectures or workshops from a robot?

- Yes
- No
- Neutral

21. In your opinion, does receiving information from a teaching robot increase self-confidence more than in a traditional classroom?

- Yes
- No
- Neutral

22. If you had the opportunity to work in a team that included a robot as a participant, would you agree to join?

- Yes
- No
- Neutral

23. Would you like to learn about R/AI in the future?

- Yes
- No
- Neutral

24. Has the time come for students, doctors and individuals working in the university to accept R/AI techniques?

- Yes
- No
- I don’t know

25. Do you feel that application of R/AI could enhance your clinical practice?

- Yes
- No
- I don’t know

26. Do you think that there is a need to switch to a secure digital environment using artificial intelligence applications and create a healthcare system with all the latest technologies?

- Yes
- No
- I don’t know
