# Supplementary material for: Knowledge, perception, and attitude of Egyptian dental students toward the role of robotics and artificial intelligence in dental practices - a cross-sectional study
Source: BMC Oral Health. 2025 May 21;25:747. doi: 10.1186/s12903-025-06077-0 (PMC12093707; doi:10.1186/s12903-025-06077-0)
Supplement: Supplementary file 2 — Supplementary Material 2 [file 12903_2025_6077_MOESM2_ESM.docx]

**Table (7): The scores of knowledge toward robotics and artificial intelligence.**

| **Variables** | **knowledge about robotics and artificial intelligence** | | | |
| --- | --- | --- | --- | --- |
|  | **Mean** | **SD** | **Median** | **Range** |
| **Q2** | 0.41 | 0.49 | 0.00 | 1.00 |
| **Q3** | 0.57 | 0.50 | 1.00 | 1.00 |
| **Q4** | 0.70 | 0.46 | 1.00 | 1.00 |
| **Q5** | 0.81 | 0.40 | 1.00 | 1.00 |
| **Q6** | 0.73 | 0.44 | 1.00 | 1.00 |
| **Q7** | 0.69 | 0.46 | 1.00 | 1.00 |
| **Total** | 0.65 | 0.28 | 0.67 | 1.00 |

.

**Table (8): The scores of Perception toward robotics and artificial intelligence.**

| **Variables** | **Perception toward robotics and artificial intelligence** | | | |
| --- | --- | --- | --- | --- |
|  | **Mean** | **SD** | **Median** | **Range** |
| **Q8** | 0.72 | 0.45 | 1.00 | 1.00 |
| **Q9** | 0.65 | 0.48 | 1.00 | 1.00 |
| **Q10** | 0.77 | 0.42 | 1.00 | 1.00 |
| **Q11** | 0.54 | 0.50 | 1.00 | 1.00 |
| **Q12** | 0.81 | 0.39 | 1.00 | 1.00 |
| **Q13** | 0.82 | 0.38 | 1.00 | 1.00 |
| **Q14** | 0.67 | 0.47 | 1.00 | 1.00 |
| **Q15** | 0.80 | 0.40 | 1.00 | 1.00 |
| **Q16** | 0.62 | 0.49 | 1.00 | 1.00 |
| **Total** | 0.71 | 0.25 | 0.78 | 1.00 |

**Table (9): The scores of Attitude toward robotics and artificial intelligence.**

| **Variables** | **Attitude toward robotics and artificial intelligence** | | | |
| --- | --- | --- | --- | --- |
|  | **Mean** | **SD** | **Median** | **Range** |
| **Q17** | 0.56 | 0.50 | 1.00 | 1.00 |
| **Q18** | 0.51 | 0.50 | 1.00 | 1.00 |
| **Q19** | 0.73 | 0.45 | 1.00 | 1.00 |
| **Q20** | 0.31 | 0.47 | 0.00 | 1.00 |
| **Q21** | 0.32 | 0.47 | 0.00 | 1.00 |
| **Q22** | 0.59 | 0.49 | 1.00 | 1.00 |
| **Q23** | 0.74 | 0.44 | 1.00 | 1.00 |
| **Q24** | 0.69 | 0.47 | 1.00 | 1.00 |
| **Q25** | 0.74 | 0.44 | 1.00 | 1.00 |
| **Q26** | 0.73 | 0.44 | 1.00 | 1.00 |
| **Total** | 0.59 | 0.28 | 0.60 | 1.00 |
